# Improving Nursing Students’ Critical Thinking Disposition Through a Gamified Flipped Classroom: A Quasiexperimental Study

**DOI:** 10.1155/nrp/5074543

**Published:** 2026-09-16

**Authors:** Mohammad Sadegh Dehghan, Mehdi Birjandi, Maryam Bagheri

**Affiliations:** ^1^ Department of Medical Surgical Nursing, School of Nursing and Midwifery, Isfahan University of Medical Sciences, Isfahan, Iran, mui.ac.ir; ^2^ Department of Biostatistics and Epidemiology, School of Health and Nutrition, Lorestan University of Medical Sciences, Khorramabad, Lorestan, Iran, lums.ac.ir; ^3^ Nursing and Midwifery Care Research Centre, Department of Medical-Surgical Nursing, School of Nursing and Midwifery, Isfahan University of Medical Sciences, Isfahan, Iran, mui.ac.ir

**Keywords:** critical thinking disposition, flipped classroom, gamification, nursing student

## Abstract

**Background:**

Combining gamification with the flipped classroom as a novel teaching method for enhancing the learning process has theoretical advantages.

**Aim:**

This study aimed to investigate the effect of the gamified flipped classroom method on the critical thinking disposition of undergraduate nursing students at Isfahan University of Medical Sciences.

**Methods:**

This study was a single‐group, quasiexperimental design with a pretest and a posttest. This study was conducted on 60 fourth‐semester nursing students. The intervention consisted of a gamified flipped classroom, with the preclass component delivered on a virtual learning management system with games and the in‐class component gamified in face‐to‐face classes. The Persian version of the California Critical Thinking Disposition Inventory (CCTDI) was used.

**Results:**

A statistically significant improvement was observed in overall critical thinking disposition and in the subscales of open‐mindedness, analyticity, and maturity following the implementation of the gamified flipped classroom (*p* = 0.001). Effect sizes were moderate to strong (95% CI: −0.71 to −0.47).

**Conclusion:**

This study suggests that integrating a gamified flipped classroom with in‐class games may enhance nursing students’ disposition toward critical thinking.

## 1. Introduction

Today’s clinical environments are marked by patient complexity and diversity, requiring nursing graduates to develop cognitive skills to manage care challenges [1]. Promoting critical thinking among nursing students is a professional requirement that impacts the quality of care and patient outcomes [2]. Critical thinking, as defined by Facione [3], includes the skills of interpretation, analysis, evaluation, explanation, self‐regulation, and inference. However, developing critical thinking skills also requires sufficient inclination and willingness to develop and apply these skills [3]. These tendencies and inclinations need to be continually examined and reinforced. Flipped classroom and gamification are educational strategies that have been suggested to improve students’ critical thinking [4, 5].

The flipped classroom consists of two main components: technology‐based preclass activities and in‐class activities that apply the acquired knowledge, and its goal is to engage students with course concepts [6]. Despite the potential benefits of the flipped classroom, this method has limitations such as rejection by some students who consider it to impose extra work on them, inadequate quality of produced content, decreased motivation and less student engagement, and the need to prepare students for e‐learning [7, 8]. Also, the results of studies on the impact of flipped classrooms on critical thinking are sometimes inconsistent [9–11]. One of the proposed solutions to enhance the effectiveness of flipped classrooms is to combine the flipped classroom with gamification [12]. In other words, it is recommended that incorporating game elements—such as point scoring, leaderboards, and rewards—can serve as a key factor in improving the learning process in the flipped classroom [13].

A conceptual linkage between gamification, flipped learning, and critical thinking disposition can be explained through their combined influence on engagement, motivation, and cognitive processes. Integrating gamification into flipped learning environments has been shown to enhance students’ engagement, motivation, and readiness for learning [12, 14, 15], which are prerequisites for the development of critical thinking disposition [16, 17]. Gamification further reinforces this process by promoting emotional and cognitive engagement through structured elements such as feedback, challenge, and progression [13, 18]. Increased engagement has been associated with deeper information processing and reflective thinking, both of which contribute to the development of critical thinking and critical thinking disposition [19–21].

The use of gamified flipped classrooms has attracted increasing attention in recent years. However, few studies have examined the impact of using gamified flipped classrooms on critical thinking disposition. Furthermore, studies in this area have focused on integrating gamification with in‐class learning sessions in flipped classrooms, while the potential of integrating gamification into flipped classrooms for extracurricular learning activities warrants further investigation. Therefore, this study aimed to determine and compare the mean scores of critical thinking disposition and its subscales before and after a gamified flipped classroom intervention in nursing students. The following research questions were formulated:

• RQ1: Does the mean total critical thinking disposition score differ significantly before and after the intervention in the participants?
• RQ2: Do the mean scores of the critical thinking disposition subscales (truth‐seeking, open‐mindedness, analytical ability, information systematic ability, inquisitiveness, self‐confidence, and maturity) differ significantly before and after the intervention in the participants?

## 2. Materials and Methods

This study employed a single‐group, pretest–posttest, quasiexperimental design [22], which was conducted in the second semester of 2024 at the Faculty of Nursing, Isfahan University of Medical Sciences. A census sampling method was employed, whereby all eligible students from the target population (*N* = 72) were invited to participate. The required sample size was initially estimated using the following formula: ($n={\left(z_{12-\alpha /}+z_{1-\beta }\right)}^{2}\times s^{2}/d^{2}$). Given the absence of prior studies employing the California Critical Thinking Disposition Inventory (CCTDI) in a comparable context, parameter estimates were derived from a methodologically similar study that examined the effect of a flipped classroom on critical thinking disposition among nursing students using a validated instrument [23]. Considering *α* = 0.05, *β* = 0.20 (power = 80%), standard deviation (*s* = 10), and a minimum meaningful difference (*d* = 4), the required sample size was calculated to be 49 participants. A total of 60 participants were included in the final analysis, which exceeded the minimum required sample size and thereby ensured adequate statistical power for the study. Inclusion criteria for the study included registration and enrollment in the theoretical course “Adult Health Nursing 2,” stable access to the internet and technological tools (such as a mobile phone or computer), and students’ self‐declaration of willingness to participate in the study. Exclusion criteria included individuals’ lack of desire to continue participating in the study, failure to complete or incomplete completion of questionnaires, and lack of active participation in two consecutive sessions during the teaching. Students participated in 5 class sessions held over 5 weeks (one class session per week). Of the 72 students, 6 students were excluded from the study due to noncompletion or incomplete completion of the questionnaire, and 6 students were excluded due to nonparticipation in more than 2 sessions of the gamified flipped classroom sessions. A total of 60 students (83.3%) participated in the study.

### 2.1. Data Collection Tools

Data were collected using a questionnaire on students’ demographic information including age, gender, grade point average, marital status, and residence status (local or dormitory). Also, the Persian version of the CCTDI [24] was used to collect data [25].

The total score of this questionnaire comprises seven dimensions: truth‐seeking (12 items), open‐mindedness (12 items), analytical ability (11 items), information systematic ability (11 items), inquisitiveness (10 items), self‐confidence (9 items), and maturity (10 items). The questionnaire employs a six‐point Likert scale: *strongly agree*, *mostly agree*, *agree*, *disagree*, *mostly disagree*, and *strongly disagree*. The minimum score on this test is 70, and the maximum score is 420. The minimum score for each dimension of critical thinking disposition is 10, and the maximum is 60. A total score below 210 indicates a negative disposition, a score between 210 and 279 indicates an unstable disposition, a score between 280 and 350 indicates a positive disposition, and a score above 350 indicates a strong disposition. In the study, the analysis focused on mean scores and their comparisons across variables. Therefore, the cutoff points were not used for categorizing participants but are presented only to provide a general framework for interpreting the scores.

The face and content validity of this questionnaire have been confirmed by ten faculty members and experts in nursing, psychology, and management [26]. In addition, the validity and reliability of the Persian version of this questionnaire for nursing students have been confirmed, and a Cronbach’s alpha coefficient of 0.9 was calculated [25].

### 2.2. Intervention Implementation

In this study, the intervention was carried out using a game‐based flipped classroom approach over five sessions as part of the “Adult and Elderly Nursing 2” course unit, focusing on nursing care for patients with renal and urinary disorders. This lesson focused on identifying symptoms, signs, diagnostic tests, treatment, and complications, as well as presenting nursing care principles—from health promotion and disease prevention to disability management—implemented through the nursing process.

In designing this study, the primary framework for delivering the educational content was based on the flipped classroom model. Preclass content and learning activities were delivered online in a gamified format through an open‐source learning management system called Moodle. This web‐based system was accessible on all devices commonly used by students, including smartphones, tablets, laptops, and personal computers. Additionally, it supported gamification features such as badges, scores, and leaderboards. The online educational content and its supplementary materials—which included standardized questions for each topic at various difficulty levels, clinical scenarios (virtual simulated patients with medical history, clinical tests, and diagnostic documentation), and educational videos—were prepared and approved by the instructors. Then, using the tools and plugins available in the Moodle system, these were organized as separate gamified activities for each session. To systematically gamify this instructional content, we implemented the the gamification process based on the Huang and Hew model, which applies motivational gamification theories in practice. According to Huang and Hew, effective gamification requires aligning motivational elements with educational objectives. In our implementation, clear goals were set through defined milestones for required activities; autonomy was supported by Bloom’s taxonomy‐based questions at easy, intermediate, and hard levels, along with mandatory and supplementary tasks offering extra points and multiple pathways in clinical scenarios; challenges were introduced by ranking top performers and incorporating group evaluation during in‐person sessions; immediate feedback was provided after practice questions and in final reviews; and collaboration was fostered by dividing students into small groups to tackle complex cases [27].

In this study, the gamified content was provided to 5 faculty members who were experts in the field of kidney diseases and urology and 5 nurses with a background in nephrology and urology departments. Content and activities that had a validity of less than 0.8 were individually eliminated and merged into other activities. Then, scoring was performed again for each activity, and content validity was confirmed. After that, the content was presented to 2 experts who had research experience in educational gamification, and the gamification validity was confirmed based on the EGame Flow Scale [28].

First, the research objectives and the implementation of gamification with a flipped classroom approach were explained to the students; then, during an in‐person orientation session, students were taught how to use the system and the intervention, and consent forms and critical thinking disposition questionnaires were completed.

### 2.3. Out‐of‐Class Educational Content

One week before each in‐person session, gamified content was made available through the platform for students. When a student began a session for the first time, they were required to sequentially complete the essential educational activities and earn points for each activity completed initially. In addition, optional supplementary activities were accessible from the start of the session and similarly rewarded upon their first attempt. This section was designed based on process‐oriented assessments, which are a type of logistical educational strategy. These assessments are founded on the concept of mastery through sequential learning, such that mastery of each section is a prerequisite for learning the next one [29].

#### 2.3.1. Essential Activities

(1) Students answer 10 multiple‐choice questions on prerequisite topics. A score below 70% requires a review and retake; achieving 70% or more allows them to move on. (2) Instructional video: A session‐related video—covering key concepts such as pathophysiology, diagnosis, treatment, and nursing—is made available. The video can be rewatched and its speed adjusted. (3) Practice questions: After the video, students can practice with multiple‐choice questions offered at three difficulty levels (easy: 1 point per question, medium: 2 points, hard: 3 points). Points from these are added to the total score. (4) Final examination: Students then take an examination with 10 randomly selected questions from the pretest and practice questions. Scoring at least 70% completes the session’s essential activities.

#### 2.3.2. Supplementary Activities

(1) Virtual patient simulation: In a role‐playing exercise using specialized software, students interact with a virtual patient by selecting preset questions. Exploring additional clinical data earns extra points, with more points awarded for deeper investigation. (2) Badges: Once a student accumulates 100 points, a badge is automatically awarded in the learning management system.

#### 2.3.3. In‐Class Flipped Classroom Gamification

Students form small groups (5–7 members) and begin by reviewing the online leaderboard and taking a short quiz on their preclass learning. The instructor then delivers a brief lecture to address any issues. Groups work on clinical scenarios, and through a gamified Q&A where individual responses impact group scores, the highest‐scoring group is declared the winner.

Finally, after every five sessions, students complete a questionnaire to assess their critical thinking disposition. Data analysis was performed using SPSS Version 16. The assumption of normality was evaluated using the Kolmogorov–Smirnov test and supported by graphical assessments. The results indicated no significant deviation from normality (*p* > 0.05). The results were reported at a significance level of 0.05. The magnitude of effect size for paired *t*‐tests was estimated using Cohen’s *d*, with values of 0.2, 0.5, and 0.8 interpreted as small, medium, and large effects, respectively, according to Cohen’s guidelines [30].

### 2.4. Ethical Considerations

Before starting the research, necessary permissions were obtained, and then all participants were provided with detailed information about the aims, procedures, and benefits of the study. Written informed consent was obtained from each participant before entering the study. Data were anonymized to protect the identity of the participants. Personal information and participants’ responses were kept confidential and analyzed. This project was approved by the Research Ethics Committee of School of Nursing, Management, and Rehabilitation at Isfahan University of Medical Sciences with the Ethics ID IR.MUI.NUREMA.REC.1402.138.

## 3. Results

The average age of participants in this study was 21.83 ± 1.34 years. The majority of participants were single (96.7%). The participants’ mean GPA was 15.38 ± 1.25 (Table 1). Additionally, 84.7% of the participants attended 4 out of 5 sessions of the flipped classroom gamification out‐of‐class activities. The average number of sessions in which students actively participated in these activities was 3.96 out of 5.

**TABLE 1:** Demographic characteristics.

Variable	Category	*N*	%
Gender	Male	29	48.3
	Female	31	51.7

Marital status	Single	58	96.7
	Married	2	3.3

Residency	Native	40	66.7
	Dormitory	20	33.3

Age	Mean (SD)	21.83	(1.34)

GPA	Mean (SD)	15.38	(1.25)

The results of this study showed that the mean scores of critical thinking disposition increased following the gamified flipped classroom intervention. Statistically significant improvements were observed in the dimensions of open‐mindedness, analytical ability, and maturity (*p* ≤ 0.001), with maturity showing a moderate‐to‐large effect size (ES = −0.71). Smaller but statistically significant increases were also found in inquisitiveness (*p* = 0.032) and self‐confidence (*p* = 0.031); however, these results should be interpreted with caution due to their proximity to the conventional significance threshold. No statistically significant changes were observed in truth‐seeking (*p* = 0.065) and systematicity (*p* = 0.442). The total mean score of critical thinking disposition increased from 278.48 ± 16.78 before the intervention to 290.23 ± 18.98 after the intervention, which indicated a statistically significant improvement (*p* = 0.001) with a medium effect size (ES = −0.59) (Table 2).

**TABLE 2:** Changes in the mean score of critical thinking disposition before and after the gamified flipped classroom.

Variable	Pretest mean (SD)	Posttest mean (SD)	*p* value	Effect size	95% CI for effect size
Truth‐seeking	39.43 (4.82)	40.65 (5.71)	0.065	−0.24	(−0.5, 0.015)
Open‐mindedness	42.23 (4.56)	44.68 (5.30)	0.001	−0.47	(−0.73, −0.2)
Analytical	45.50 (4.98)	47.83 (5.03)	0.001	−0.47	(−0.73, −0.2)
Systematicity	41.97 (4.58)	42.43 (5.84)	0.442	−0.10	(−0.35, 0.15)
Inquisitiveness	36.60 (4.56)	37.82 (4.94)	0.032	−0.28	(−0.54, −0.03)
Maturity	35.15 (3.65)	37.80 (4.35)	0.001	−0.71	(−0.99, −0.42)
Self‐confidence	37.60 (5.36)	39.02 (5.68)	0.031	−0.29	(−0.54, −0.03)
Total score	278.48 (16.78)	290.23 (18.98)	0.001	−0.59	(−0.85, −0.31)

## 4. Discussion

Gamification in nursing education enhances cognitive skills by engaging students in scenario analysis, decision‐making, and problem‐solving. It also provides a risk‐free environment for learning from errors [31]. The findings of this study suggest that the use of a gamified flipped classroom was associated with improvements in nursing students’ critical thinking disposition. Following the intervention, students showed higher mean scores in critical thinking disposition, with a moderate effect size. Statistically significant increases were observed in several dimensions, including open‐mindedness, analytical ability, inquisitiveness, maturity, and self‐confidence, with effect sizes ranging from small to moderate, and a relatively larger effect observed for maturity. However, no statistically significant changes were found in truth‐seeking or systematicity.

A statistically significant increase was observed in open‐mindedness, with a moderate effect size. The game elements in the flipped classroom encouraged students to explore different ideas and scenarios. This helped them learn about each idea before deciding if it was good or bad. The games also made them join in more during class discussions, which helped them become more open to new ideas. Additionally, the playful environment reduced their fear of making mistakes and tempted them to try new things, thereby strengthening their open‐mindedness [32].

The observed increase in the analytical dimension can be explained by the problem‐solving activities—such as clinical scenarios—that require students to assess complex cases and evaluate various solutions. Moreover, gamified interactive learning deeply engages students with the material, fostering a thorough understanding of key concepts and improving their capacity to analyze information. Immediate feedback further enables them to identify areas for improvement and continuously refine their analytical skills [33].

One possible explanation for the observed increase in inquisitiveness may be related to the interactive and engaging nature of the content, which can stimulate students’ curiosity [34]. Additionally, activities related to clinical scenarios may enhance active learning and encourage students’ exploration. However, given the absence of a control group, the observed improvements should be interpreted with caution. It is possible that factors such as maturation or testing effects, rather than the intervention alone, contributed to the increase in students’ inquisitiveness. The observed increase in maturity could potentially be explained by the emphasis on students’ responsibility for their own learning within this method [34, 35]. Also, the shift toward a more playful and engaging classroom environment may be associated with improvements in student self‐confidence. By potentially reducing the fear of failure and providing a supportive space for experimentation and learning, this approach may encourage students to take risks and engage more actively with challenges [34, 36]. These experiences could, in turn, be linked to increased self‐confidence. Furthermore, in‐person group activities may contribute to collaboration and peer support. Working toward shared goals can foster a sense of belonging and mutual encouragement, which may also be associated with self‐confidence [35, 37]. The observed increase in critical thinking disposition may be associated with the integrated use of a flipped classroom and gamification. This combined approach likely promotes a more active and student‐centered learning environment by requiring learners to engage with content before class and participate in in‐class, problem‐based activities [12, 14, 34]. Gamification may further enhance motivation and sustained engagement, thereby encouraging deeper involvement in learning processes [13, 18]. Increased engagement and motivation have been identified as important contributors to critical thinking disposition development [16, 17]. Accordingly, the synergy between these strategies may provide repeated opportunities for higher‐order thinking [4, 5, 34], which could account for the improvement observed in critical thinking disposition. Despite these findings, several limitations should be considered. The single‐group pretest–posttest design and the absence of a control group limit causal inference regarding the intervention’s effectiveness. Maturation and testing effects may also have influenced the results, alongside factors such as increased motivation, concurrent coursework, or external learning experiences.

Comparative studies are recommended to identify the most effective gamification strategies and their optimal integration within flipped classroom frameworks. Future research should further examine the effects of gamified flipped classrooms on the development of clinical competencies, patient care outcomes, and their integration with emerging technologies to enhance educational effectiveness.

## 5. Conclusion

This study suggests that gamification is a valuable complement for enhancing nursing students’ active participation in the flipped classroom learning process. The combined use of these two techniques may foster a stronger critical thinking disposition among nursing students.

### 5.1. Limitations

This study was conducted among second‐year undergraduate nursing students using a single‐group pretest–posttest design, which limits the generalizability of the findings. The absence of a control group reduces the ability to make causal inferences regarding the observed changes, as improvements cannot be attributed solely to the intervention. In addition, testing effects may have influenced the results, as exposure to the pretest could have affected participants’ posttest performance. Accordingly, the findings should be considered preliminary.

## Author Contributions

Mohammad Sadegh Dehghan: data curation, investigation, methodology, resources, validation, writing–original draft, and writing–review and editing. Mehdi Birjandi: formal analysis, methodology, validation, visualization, writing–original draft, and writing–review and editing. Maryam Bagheri: conceptualization, funding acquisition, investigation, methodology, project administration, supervision, validation, visualization, roles/writing–original draft, and writing–review and editing.

## Funding

This study was supported by the Isfahan University of Medical Sciences (grant number: 3402386).

## Disclosure

This study was extracted from the thesis of Mr. Mohammad Sadegh Dehghan, which was conducted at Isfahan University of Medical Sciences.

## Conflicts of Interest

The authors declare no conflicts of interest.

## Data Availability

The data supporting the findings of this study are available from the corresponding author upon reasonable request.
